# Further insights into influence factors of hypertension in older patients with obstructive sleep apnea syndrome: a model based on multiple centers

**DOI:** 10.1007/s40520-025-02986-w

**Published:** 2025-03-27

**Authors:** Libo Zhao, Xin Xue, Yinghui Gao, Weihao Xu, Zhe Zhao, Weimeng Cai, Dong Rui, Xiaoshun Qian, Lin Liu, Li Fan

**Affiliations:** 1 Cardiology Department of the Second Medical Center & National Clinical Research Center for Geriatric Diseases, Chinese PLA General Hospital, Beijing, 100853 China; 2 Department of Respiratory and Critical Care Medicine, Affiliated Hospital of Yan’an University, Yan’an, 716000 China; 3 Sleep Center, Peking University International Hospital, Beijing, 102206 China; 4 Cardiology Department of Guangdong Provincial People’s Hospital, Guangzhou, 510080 China; 5 Graduate School, Medical School of Chinese PLA, Beijing, 100853 China; 6 Department of Respiratory and Critical Care Medicine of the Second Medical Center & National Clinical Research Center for Geriatric Diseases, Chinese PLA General Hospital, Beijing, 100853 China

**Keywords:** Hypertension, OSAS, Older, Model, Nomogram, Scoring system

## Abstract

**Objective:**

To construct a novel model or a scoring system to predict hypertension comorbidity in older patients with obstructive sleep apnea syndrome (OSAS).

**Methods:**

A total of 1290 older patients with OSAS from six tertiary hospitals in China were enrolled. The sample was randomly divided into a modeling set (80%) and validation set (20%) using a bootstrap method. Binary logistic regression analysis was used to identify influencing factors. According to the regression coefficients, a vivid nomogram was drawn, and an intuitive score was determined. The model and score were evaluated for discrimination and calibration. The *Z*-test was utilized to compare the predictive ability between the model and scoring system.

**Results:**

In the multivariate analysis, age, body mass index (BMI), apnea–hypopnea index (AHI), total bilirubin (TB), high-density lipoprotein cholesterol (HDL-C), and fasting blood glucose (FBG) were significant predictors of hypertension. The area under the receiver operating characteristic curve of the model in the modeling and validation sets was 0.714 and 0.662, respectively. The scoring system had predictive ability equivalent to that of the model. Moreover, the calibration curve showed that the risk predicted by the model and the score was in good agreement with the actual hypertension risk.

**Conclusions:**

This accessible and practical correlation model and diagram can reliably identify older patients with OSAS at high risk of developing hypertension and facilitate solutions on modifying this risk most effectively.

**Supplementary Information:**

The online version contains supplementary material available at 10.1007/s40520-025-02986-w.

## Introduction

Sleep accounts for approximately one-third of an individual’s life, and adverse reactions that occur during sleep can have major health consequences. Obstructive sleep apnea syndrome (OSAS) is a common and potentially fatal clinical condition caused by a narrowing or repeated collapse of the throat during sleep [1]. A global study shows that nearly a billion people are affected by this syndrome, with a prevalence exceeding 50% in some countries [2]. The immediate consequences of airway collapse are intermittent hypoxia, repeated awakenings, and increased respiratory effort, leading to secondary sympathetic activation, oxidative stress, and systemic inflammation [3]. These mechanisms, in turn, contribute to increased vascular tension and blood pressure. In middle-aged and older populations, the detection rate of OSAS increases with the severity of hypertension [4].

OSAS and hypertension often coexist, and the prevalence of OSAS in hypertensive patients ranges from 30 to 50%. In contrast, in patients with refractory hypertension, this rate is further increased to 70% [5]. To date, OSAS is generally accepted to be the most common secondary associated disorder [6]. Although OSAS and hypertension often appear simultaneously, associations between them can either reflect causality or indicate different endpoints with similar risk factors. In general, they are closely related and both are risk factors for adverse cardiovascular and cerebrovascular events [7, 8]. For example, one study suggested that OSAS may lead to more unstable coronary plaques in patients with coronary heart disease [9]. Another study showed that OSAS could affect left ventricular diastolic function independent of other possible factors [10]. Furthermore, the blood pressure wave pattern of OSAS-related hypertension is mostly non-dipper-type, which is associated with a more significant risk and worse severity of target organs damage than dipper-type hypertension [11]. The prevalence of both diseases increases with age, and limited information is available about the factors affecting OSAS-related hypertension in older Chinese adults. Therefore, studying and preventing OSAS-related hypertension are of great significance.

This study attempts to establish a predictive model for OSAS-related hypertension to help to indicate an older individual’s risk of developing hypertension. Through effective subjective prevention and treatment, the damage to important organs caused by OSAS and hypertension can be delayed, and the occurrence of serious complications can be reduced.

## Materials and methods

### Study population

In this cross sectional study, subjects were consecutive patients diagnosed with OSAS by the Sleep Center of the People’s Liberation Army (PLA) General Hospital, Peking University International Hospital, Peking University People’s Hospital, Beijing Chaoyang Hospital, the 960th Hospital of the PLA and the Affiliated Hospital of Gansu University of Chinese Medicine in China from January 2015 to October 2017. A total of 1290 older patients with OSAS were enrolled in this study, all of whom were diagnosed by polysomnography (PSG) examination [12]. Data from the first diagnosis of OSAS were obtained from patients with single or multiple visits and hospitalizations. The study was conducted in strict accordance with the Declaration of Helsinki and was approved by the Medical Ethics Committee of the PLA General Hospital (No. S2019-352-01). We managed the confidentiality of patient information and related case data. Patients or the public were not involved in the design, or conduct, or reporting, or dissemination plans of our research, we collected and used only the clinical data that were generated in the normal course of their care.

The apnea–hypopnea index (AHI) was calculated as the number of apnea–hypopnea events per hour and can be used to determine the severity of OSAS. AHI ≥ 5 events/h with obstructive apnea events (accounting for more than 50% of respiratory events) was considered a diagnosis of OSAS [13]. OSAS was classified into mild (5 ≤ AHI < 15), moderate (15 ≤ AHI < 30) and severe (AHI ≥ 30) according to the value of AHI [14]. All suitable older patients were enrolled according to the above criterion. Comparatively, patients were excluded according to the following criteria: (1) Age < 60 years old; (2) Simple snoring; (3) No PSG monitoring was performed; (4) Daytime resting oxygen saturation below 90% due to pulmonary disease; (5) Patients with severe heart, kidney and other organ failure, or other cachexia; (6) Secondary hypertension from other causes, such as Cushing’s syndrome (using available medical records for screening).

### Data collection

All the indicators recorded in the study were the corresponding indicators when the patients were newly diagnosed with OSAS and did not receive intervention for it. Demographic data (e.g., sex and age), laboratory indicators, sleep-apnea data, and history of related diseases were collected from outpatient visits and inpatient medical records using a self-designed Microsoft Excel spreadsheet. Several laboratory variables were considered. The blood samples used to test the total bilirubin and other biochemical indicators were taken in the fasting state of patients in the morning. All participants underwent overnight polysomnography by using portable sleep monitoring instrument (Compumedics, Melbourne, Australia). Tea, coffee and sedative hypnotic drug were forbidden before bedtime. PSG monitoring includes electroencephalogram, electrooculography, transnasal airflow, and nocturnal pulse oxygen saturation, etc. [15]. All clinical and laboratory variables were carefully recorded and reviewed by 2 or 3 specialists.

Outcome indicator: OSAS-related hypertension can be diagnosed when OSAS is associated with hypertension [16]. Diagnostic criteria for hypertension [17]: In the absence of antihypertensive drugs, the patient’s blood pressure was measured three times on different days with systolic blood pressure (SBP) ≥ 140 mm Hg and/or diastolic blood pressure (DBP) ≥ 90 mm Hg; Mean 24 h ambulatory blood pressure ≥ 130/80 mm Hg; A definite history of hypertension diagnosis and treatment or antihypertensive medication. Avoid drinking tea and drinks containing caffeine and have not smoked for at least 30 min before blood pressure measurement. In a quiet, relaxed environment, after the patient had rested for 5 min in the sitting position, the upper limb blood pressure was measured by a qualified medical staff using a standard electronic sphygmomanometer with calibration.

### Statistical analysis

The subjects were divided into two groups according to the presence or absence of hypertension. Data were first tested for normality and homogeneity of variance. For continuous variables, the independent group *t* test was used for normally distributed data and the Mann–Whitney test was used for non-normally distributed data. For categorical variables, X^2^ or Fisher’s exact test was used to compare proportions between groups. A total of 1290 patients were randomly divided into a modeling set and a validation set consisting of 1032 and 258 patients, respectively, in a 4:1 ratio. The modeling set was used to develop the predictive nomogram and scoring system, and the validation set was used to evaluate their performance.

We identified the optimal variables that were statistically different between groups and did not have collinearity, and then established binary logistic regression models [18]. Thereafter, it was used to establish the prediction model of hypertension, and the nomogram was generated. The relevant influencing factors were stratified according to clinical significance and optimal cut-off value, and the corresponding score was calculated according to the regression coefficient *β* value of each variable in the newly obtained prediction model. Then, the hypertension risk score of each subject was calculated, and the scoring system was established. In the modeling set and the validation set, the discrimination of the prediction model and the scoring system was evaluated by the area under the receiver operating characteristic (ROC) curve, and the sensitivity, specificity, positive predictive value, negative predictive value and other indicators were used to analyse the accuracy. In addition, the consistency of the prediction between the two sets, the calibration evaluation, was completed by smooth curve fitting test or Hosmer–Lemeshaw test (H–L test) [19].

The statistical analyses were performed using the SPSS (version 25.0, SPSS Inc., Chicago, IL, United States) and the EmpowerStats software (www.empowerstats.net, X&Y Solutions Inc., Boston, Massachusetts, USA) [20, 21]. All statistical analyses were two-tailed, and a *P* < 0.05 was considered to be statistically significant.

## Results

### Basic information about variables

A total of 1290 subjects diagnosed with OSAS by PSG were enrolled in this study, including 798 males (61.86%). The median age was 66 years (range, 60–96 years). There were 575 patients (44.57%) with severe OSAS; 538 (41.71%) with overweight; 422 (32.71%) with obesity; and 835 (64.73%) with hypertension.

All individuals were grouped according to the presence or absence of hypertension, and the variables were compared between the two groups. The proportion of males and cases of severe OSAS, cerebrovascular accidents, coronary heart disease, diabetes, and other diseases in the hypertensive group were significantly higher than those in the non-hypertensive group. The age, body mass index (BMI), AHI, and percentage of time during sleep when SpO_2_ is below 90% of total sleep time (T90) of the patients were also higher in the hypertension group, and the differences were statistically significant (all *P* < 0.05). There were also significant differences between both two groups in clinical indicators such as total bilirubin (TB), high-density lipoprotein cholesterol (HDL-C), creatinine, and uric acid (all *P* < 0.05, Table 1).

**Table 1:** Baseline characteristics of patients with OSAS were included according to the presence or absence of hypertension

Indicators	Without hypertension	With hypertension	U or X^2^	*P* value
Number of cases	455	835		
Age (years)	65 (62, 70)	66 (63, 72)	216,677.5	<0.001
BMI (kg/m^2^)	25.28 (22.81, 27.53)	27.06 (24.69, 29.73)	229,997.0	<0.001
Waistline (cm)	89.6 ± 11.9	95.1 ± 10.7	–	<0.001
*Sleep-related indicators*				
AHI, events (h^−1^)	22.35 (11.45, 36.05)	23.2 (12.75, 41.75)	202,043.5	0.001
ODI, events (h^−1^)	14.75 (5.35, 27.45)	17.20 (7.60, 35.90)	185,180.0	0.001
Mean time of apnea (s)	23.0 (18.0, 29.0)	23.0 (19.0, 28.6)	48,812.0	0.552
Longest time of apnea (s)	56.0 (41.0, 79.0)	57.3 (42.0, 83.0)	115,778.5	0.329
MSpO_2_ (%)	93.0 (92.0, 94.1)	93.0 (91.0, 94.0)	170,068.0	0.077
LSpO_2_ (%)	80.0 (74.0, 84.0)	78.0 (68.0, 83.0)	165,136.0	0.007
Tsat90 (min)	5.68 (0.82, 34.97)	15.88 (2.60, 67.99)	192,176.0	<0.001
T90 (%)	3.61 (0.71, 11.33)	5.05 (1.20, 17.83)	158,711.0	<0.001
*Blood indicators*				
TB (μmol/L)	12.80 (9.70, 17.10)	11.20 (8.60, 14.40)	58,583.0	<0.001
DB (μmol/L)	4.00 (3.10, 5.50)	3.70 (2.80, 4.90)	60,670.0	0.004
HDL-C (mmol/L)	1.21 (1.04, 1.46)	1.07 (0.91, 1.28)	46,442.5	<0.001
Creatinine (μmol/L)	66.25 (59.45, 77.85)	73.00 (61.20, 85.00)	84,058.5	0.002
Uric acid (μmol/L)	314.55 (257.00, 371.45)	342.00 (285.00, 403.70)	82,327.5	0.001
FBG (mmol/L)	5.39 (4.93, 6.08)	5.83 (5.18, 6.75)	92,261.0	<0.001
MCH (g/L)	338.5 (331.0, 353.0)	338.0 (328.0, 346.0)	92,983.5	0.061
Hemoglobin (g/L)	138.0 (130.0, 147.0)	137.0 (127.0, 148.0)	85,685.0	0.513
*Classification indicators*				
Male, *n* (%)	259 (56.92)	539 (64.55)	7.263	0.007
Ethnic non-Han, *n* (%)	14 (3.80)	32 (4.71)	0.462	0.496
Smoking, *n* (%)	96 (21.15)	196 (23.50)	0.931	0.335
Drinking, *n* (%)	32 (23.88)	93 (27.60)	0.679	0.410
Cerebrovascular accidents, *n* (%)	38 (8.52)	191 (23.32)	42.667	<0.001
Diabetes, *n* (%)	55 (12.42)	265 (32.40)	60.584	<0.001
CHD, *n* (%)	59 (12.97)	246 (29.46)	46.086	<0.001
Carotid atherosclerosis, *n* (%)	85 (23.29)	247 (36.43)	18.888	<0.001
Severe OSAS, *n* (%)	179 (40.04)	396 (48.71)	8.726	0.003

*BMI* body mass index, *AHI* the apnea–hypopnea index, *ODI* the oxygen desaturation index, *MSpO*_*2*_ the mean pulse oxygen saturation, *LSpO*_*2*_ the lowest pulse oxygen saturation, *Tsat90* night-time spent with oxygen saturation under 90%, *T90* the percentage of time during sleep when SpO_2_ is below 90% of total sleep time, *TB* total bilirubin, *DB* direct bilirubin, *HDL-C* high density lipoprotein cholesterol, *FBG* fasting blood glucose, *MCH* mean corpuscular-hemoglobin concentration, *CHD* coronary heart disease

### Influencing factors of OSAS-related hypertension

Taking the presence or absence of hypertension as the dependent variable, and age, gender, BMI, AHI, lowest SpO_2_, total bilirubin, HDL-C, uric acid, creatinine, fasting blood glucose (FBG), homocysteine, hemoglobin, and other indicators used as independent variables, excluding collinearity factors such as neck circumference and direct bilirubin (DB), we used binary logistic stepwise regression to analyze the influencing factors of OSAS-related hypertension. The results of the full analysis showed that OR of age, BMI, AHI, and FBG were >1, while OR of TB and HDL-C were <1 (protective factors), and the *P* corresponding to the above indicators were <0.05. Further subgroup analyses of subjects of different genders, ages, and degrees of obesity showed that there were different statistically significant influencing factors in each subgroup. For instance, the influence of age, BMI, and AHI on hypertension in the non-obese group (BMI < 28 kg/m^2^) was more obvious than in the obese group (BMI ≥ 28 kg/m^2^), as detailed in Supplementary Table 1.

### Establishment and validation of an early warning model for OSAS-related hypertension

The research subjects were divided into the modeling set and validation set at a 4:1 ratio. Factors with a *P* value less than 0.05 in the above analysis were included in the logistic regression analysis of older patients with OSAS-related hypertension, and the early warning model was established using a bootstrap method with resampling 500 times. The results displayed independent predictors of age, BMI, AHI, FBG, TB, and HDL-C for hypertension in patients with OSAS. Among them, FBG and HDL-C had greater impacts on hypertension, which are shown in Table 2.

**Table 2:** Multivariate regression analysis results with bootstrap method

Variate	Mean regression coefficient	Regression coefficient 2.5%	Regression coefficient 97.5%	OR (95% CI)
Age	0.0368	0.0118	0.0667	1.037 (1.012–1.069)
BMI	0.0975	0.0437	0.1567	1.102 (1.045–1.170)
AHI	0.0093	0.0006	0.0185	1.009 (1.001–1.019)
FBG	0.1724	0.0531	0.3131	1.188 (1.055–1.368)
TB	−0.049	−0.0801	−0.0219	0.952 (0.923–0.978)
HDL-C	−1.0565	−1.6898	−0.5146	0.348 (0.185–0.598)
Constants	−3.5977	−6.5182	−0.8213	0.027 (0.002–0.439)

*BMI* body mass index, *AHI* the apnea–hypopnea index, *FBG* fasting blood glucose, *TB* total bilirubin, *HDL-C* high density lipoprotein cholesterol

**Fig. 1:**
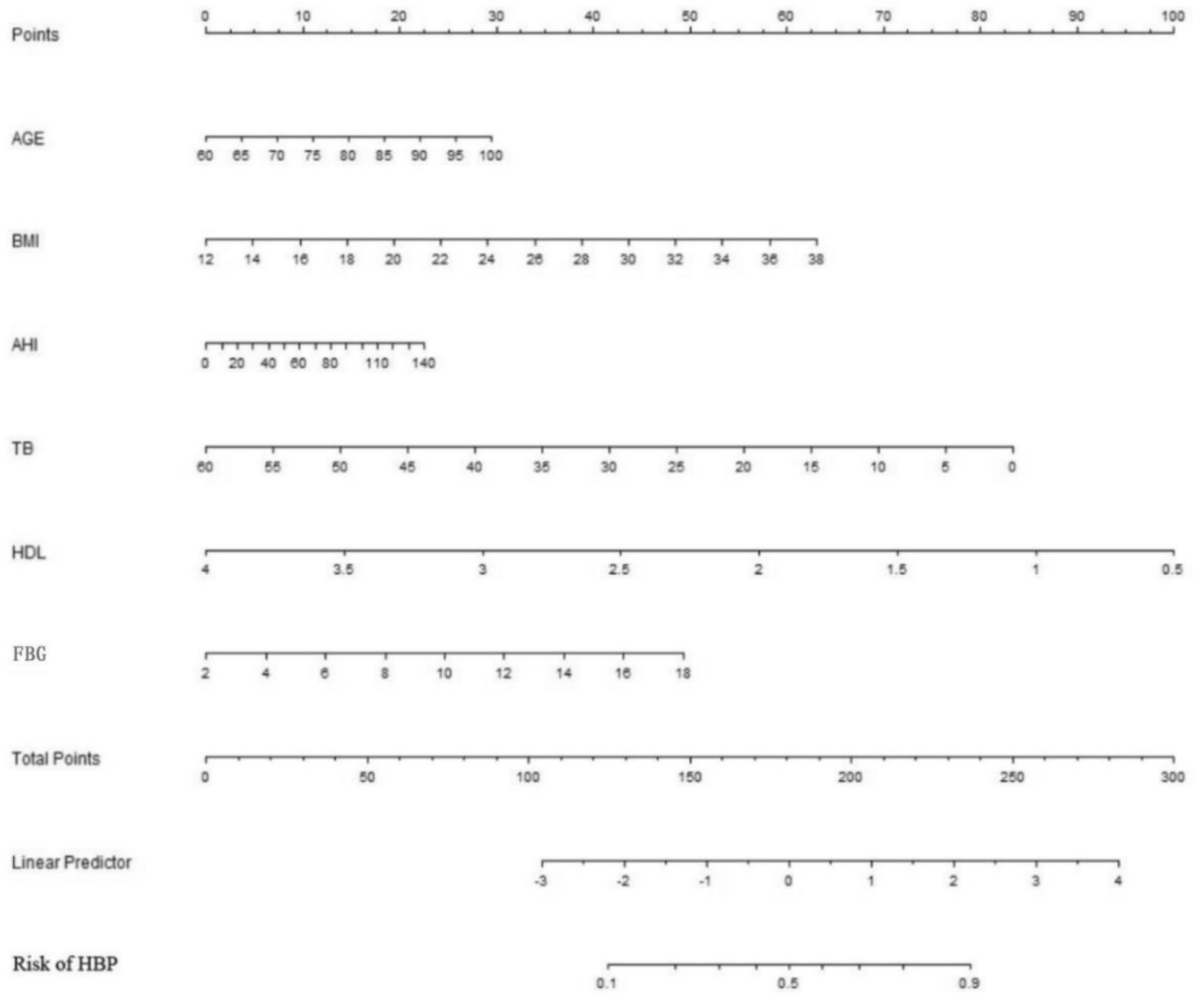
Logistic regression model corresponding to nomogram. BMI: body mass index; AHI: the apnea–hypopnea index; TB: total bilirubin; HDL-C: high density lipoprotein cholesterol; FBG: fasting blood glucose; HBP: high blood pressure

Based on the predictors obtained from the abovementioned logistic regression analysis, we created the nomogram (Fig. 1) and the following formula: logitP = −3.5977 + 0.0368X_1_ + 0.0975X_2_ + 0.0093X_3_ + 0.1724X_4_ − 1.0565X_5_ − 0.049X_6_ (X_1_: age, X_2_: BMI, X_3_: AHI, X_4_: FBG, X_5_: HDL-C, X_6_: TB). The corresponding scores of each patient’s six indicators in the nomogram were added, and the resulting sum corresponds to the risk of hypertension in patients with OSAS in the vertical direction.

### Establishment and verification of a scoring system for OSAS-related hypertension

The influencing factors of OSAS-related hypertension were stratified according to their clinical significance and cut-off value, and the score was calculated according to the regression coefficient *β* value of each variable in the abovementioned logistic regression model to establish a scoring system. To convert continuous variables into categorical variables, we used the maximum Youden index of each variable as the cut-off value. The following three continuity variables were included: FBG = 5.595 mmol/L (sensitivity: 0.590, specificity: 0.598), TB = 11.95 μmol/L (sensitivity: 0.441, specificity: 0.385), and HDL-C = 1.025 mmol/L (sensitivity: 0.542, specificity: 0.240).

In this study, each additional 10 years of age was set as 1 point, and the constant B was calculated as follows: 10 × β_age_ = 10 × 0.0368 = 0.368. After the constant B was determined, the corresponding score value of each stratification in each influencing factor was calculated according to the following formula: Points_ij_ = D/B = (W_ij_ − W_iREF_) × β_i_/B. The specific values of each variable are shown in Table 3. The theoretical individual total score ranged from −1 to 9, and the predicted probability risk of hypertension for each score can be calculated according to the following equation: P = 1/[1 + exp(0.06–0.368S)], where S represents the corresponding score.

**Table 3:** The value assignment of each variable

Indicator	Variable	Value assignment (points)	
Age	X_1_	60 ≤ Age < 70	1
		70 ≤ Age < 80	2
		80 ≤ Age < 90	3
		Age ≥ 90	4
BMI	X_2_	BMI < 24 kg/m^2^	0
		24 kg/m^2^ ≤ BMI < 28 kg/m^2^	2
		BMI ≥ 28 kg/m^2^	3
AHI	X_3_	AHI < 30 events/h	0
		AHI ≥ 30 events/h	1
FBG	X_4_	FBG < 5.555 mmol/L	0
		FBG ≥ 5.555 mmol/L	1
HDL-C	X_5_	HDL-C < 1.025 mmol/L	0
		HDL-C ≥ 1.025 mmol/L	−1
TB	X_6_	TB < 11.95 mmol/L	0
		TB ≥ 11.95 mmol/L	−1

### Discrimination

**Fig. 2:**
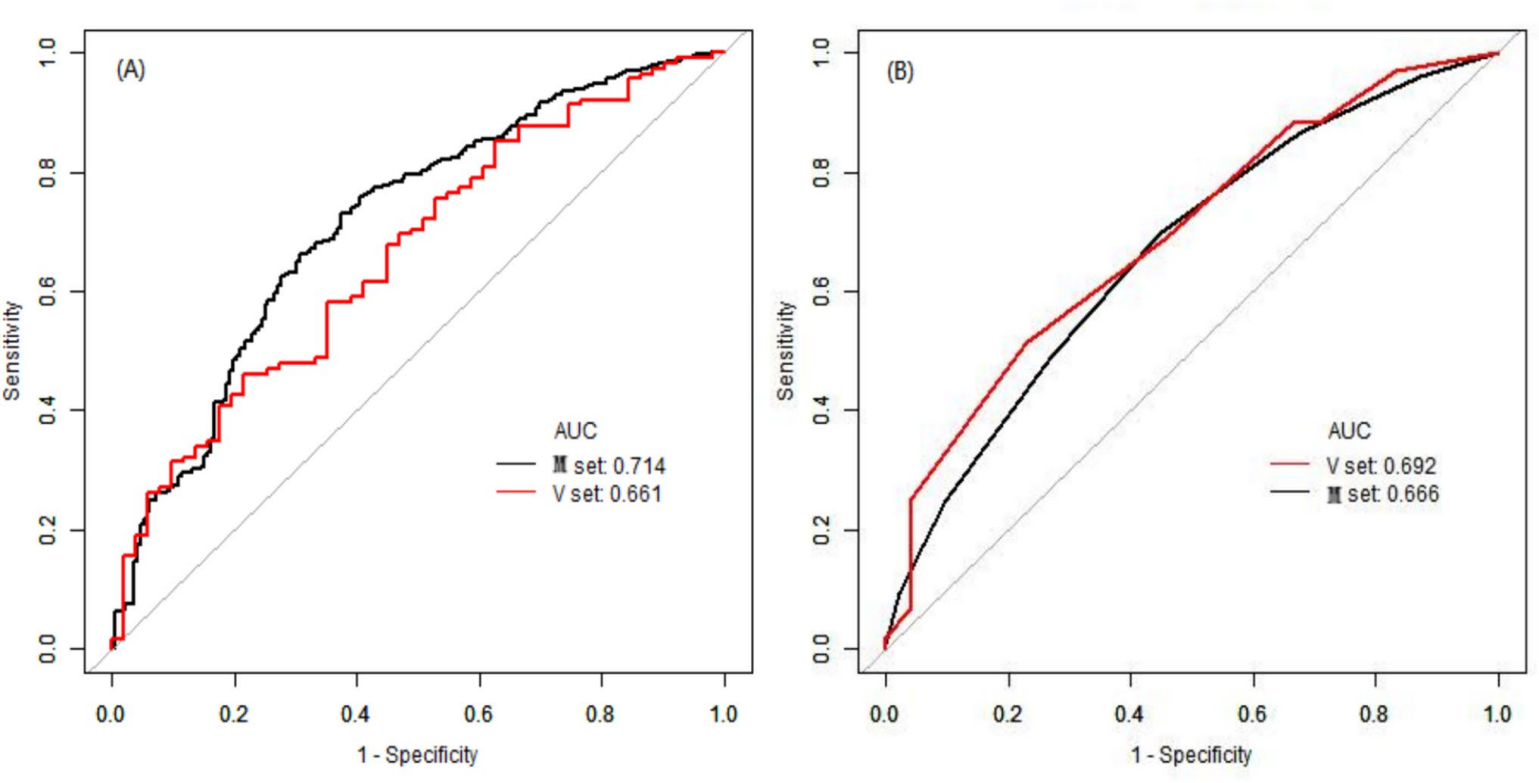
ROC curves of the modeling group and the validation group. **A** Left one, the model; **B** right one, the score. M set: the modeling group; V set: the validation group

The model: According to the obtained logistic regression model, the predicted probability of hypertension in each study subject was calculated, which was used as the diagnostic variable. The area under the ROC curve (AUC) was used to evaluate the discrimination ability of the model (Fig. 2). The AUC of the modeling and validation sets were 0.7144 and 0.6616, respectively. The specificity of the modeling and validation sets was 0.5833 and 0.7843, respectively. The sensitivity of the modeling and validation sets was 0.7733 and 0.4609, respectively (Table 5). The abovementioned data could suggest that the nomogram model had moderate discrimination ability.

The score: The individual aggregate scores of related influencing factors were calculated for each subject, and we assessed the recognition ability of the score by the uniformity of the AUC. After resampling 500 times of with the bootstrap method, similar to the above, the subjects were separated and analyzed according to a 4:1 ratio. The results showed that the AUC of the modeling set was 0.6662 (95%CI: 0.6190–0.7134, *P* < 0.05), and the AUC of the validation set was 0.6925 (95%CI: 0.6050–0.7800, *P* < 0.05) (Fig. 2; Table 5), indicating that the score had a certain evaluation ability.

### Calibration

**Fig. 3:**
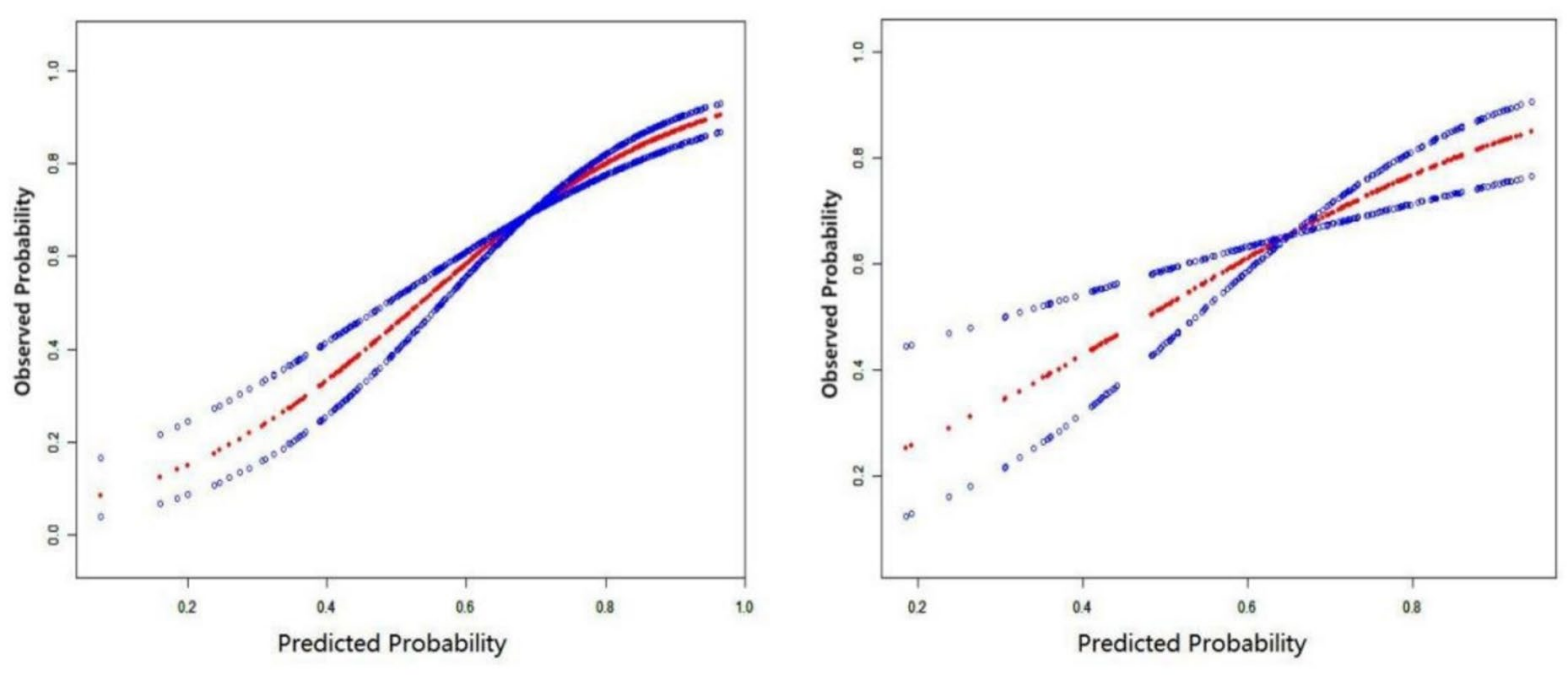
Smooth curve fitting for the modeling set (left one) and validation set (right one) in the model

The model: For accuracy, curve fitting was conducted using a standard curve-fitting tool in EmpowerStats. The results are shown in Fig. 3. The *Z* value of the modeling set was 9.42 (*P* = 0.094 > 0.05), and that of the validation set was 4.35 (*P* = 0.157 > 0.05). There was good agreement between the model-predicted risk and the actual occurrence risk in both sets.

The score: After the Hosmer–Lemeshow goodness-of-fit test was performed, the results showed that in the modeling set, χ^2^ was 2.951 (*P* = 0.707), and in the validation set, χ^2^ was 9.269 (*P* = 0.099). In other words, there was no statistically significant difference between the predicted risk and the actual observed risk in the two sets, indicating that the score agreed well with the actual occurrence risk of hypertension and had good prediction accuracy. The specific risk prediction probability is shown in Table 4.

**Table 4:** The risk prediction probability corresponding to each score in the scoring system

Scoring (point)	−1	0	1	2	3	4	5	6	7	8	9
Predicting the risk of HBP (%)	39.46	48.50	57.64	66.28	67.35	80.41	85.57	89.55	92.52	94.70	96.27

As can be seen from the table, older patient with OSAS would have a higher risk of hypertension (>80%) if his or her corresponding score is ≥4

*HBP* high blood pressure

### Comparison of evaluation ability between regression model and scoring system

The *Z*-test was used to compare the AUC between the model and the score, and the relevant formula of *Z*-test were shown in Supplementary Fig. 1. Through analysis, the results were as follows: The *Z* value of the modeling set was 1.4606 and the *Z* value of the validation set was 0.4866, both *P* > 0.05. It suggested the differences were not statistically significant, indicating that there was no significant difference between the scoring system and regression model in the risk prediction ability of older OSAS-related hypertension (Table 5).

**Table 5:** Discrimination index of modeling set and validation set

Division	AUC	95% CI	Cut-off value	Specificity	Sensitivity	DOR	PPV	NPV
*The model*								
Modeling set	0.7144	0.6700–0.7588	0.5886	0.5833	0.7733	4.7747	0.8020	0.5411
Validation set	0.6616	0.5730–0.7501	1.0921	0.7843	0.4609	3.1085	0.8281	0.3922
*The score*								
Modeling set	0.6662	0.6190–0.7134	0.6809	0.5517	0.6973	2.8348	0.7827	0.4404
Validation set	0.6925	0.6050–0.7800	0.7468	0.7708	0.5146	3.5655	0.8281	0.4253

*DOR* diagnostic odds ratio, *PPV* positive predictive value, *NPV* negative predictive value

### Illustration

A 68-year-old man with a BMI of 25.5 kg/m^2^ was identified as AHI 39 events/h after sleep breathing monitoring, FBG 5.33 mmol/L, HDL-C 1.24 mmol/L, and total bilirubin 12.25 mmol/L. By substituting the abovementioned model formula X = −3.5977 + 2.5024 + 2.4863 + 0.3627 + 0.9189 − 0.6003 − 1.310, and according to P = e^x^/(1 + e^x^), the following calculation can be obtained: P = 0.6818; According to the nomogram, the corresponding score of each index was found to be roughly Age-6, BMI-32, AHI-7, FBG-10, HDL-C-78, TB-65. Therefore, this patient had a total score of 198 and an approximately 67% probability of having hypertension; in the scoring system, the comprehensive situation of this patient should correspond to 2 points, so the probability of hypertension was 66.28%. In consequence, it can be judged that the regression model and scoring system had good and consistent evaluation effects in specific cases.

## Discussion

Hypertension is a global public health problem and an important risk factor of cardiovascular diseases [22]. Elevated blood pressure is reported in about one in four men and one in five women worldwide [23]. However, the awareness rate and the control rate of the disease have not been high [24]. OSAS is the primary secondary factor of hypertension [25], hence it is not difficult to find a higher prevalence of hypertension in patients with OSAS. According to a recent meta-analysis, it revealed a prevalence of hypertension was 44–66% in OSAS population [26]. In terms of sample size estimation in this study, the overall hypertension prevalence rate of 55% was reasonable, if the tolerance error was less than 3%, and α was 0.05, the corresponding sample size was 1056. Therefore, the subjects of this study meet the quantitative requirements and have a good representativeness. In turn, hypertension may contribute to the progression of OSAS by decreasing upper airway tone (as genoglossus activity is affected by high blood pressure) and increasing the nocturnal headend shift of fluid (because there may be volume overload associated with hypertension) [27, 28]. In brief, hypertension and OSAS together would have serious health consequences [29]. Therefore, early identification and intervention of hypertension is also an important part of helping to develop the optimal treatment plan for OSAS. In order to maximize the utilization of resources, this study selected easily measurable clinical indicators and general information of patients as predictors, and we established a prediction model for hypertension in patients with OSAS. It helps to achieve rapid screening and early warning of hypertension in patients with OSAS in a clinically heavy demand. In the following, we describe the variable choices used to build the model.

Among these statistically significant parameters, age and BMI are two well-known risk factors for hypertension. During aging, systemic blood vessels undergo structural and functional changes, including endothelial dysfunction, vessel wall thickening, and reduced dilation performance [30]. Vascular sclerosis is caused by fibrosis and remodeling of the extracellular matrix, which is related to relative increases in angiotensin II, endothelin-1 and other vasoactive substances affected by aging [31]. Some studies have also suggested that age-related hypertension may be associated with enhanced Th1 cytokine secretion, vascular remodeling, CD4^+^ T-cell infiltration in the vasculature, superoxide production, and a decreased number of nephrons [32–34]. A large-sample prospective study suggested that there was a dose effect between BMI and the relative risk of hypertension [35]. BMI is associated with extensive changes in DNA methylation that affect related genes involved in lipid metabolism, substrate transport, and inflammatory pathways, leading to hypertension and other adverse clinical consequences [36].

Aging and obesity not only increase the risk of hypertension but also decrease the muscle tone of the respiratory tract and increase the adipose tissue in the oropharynx, which increase respiratory resistance and can easily lead to OSAS [37, 38]. Vicini et al. [39] reported that the severity of OSAS increases with aging. The AHI represents the severity of OSAS. Furthermore, the higher the AHI value is, the longer the duration of hypoxia at night. Intermittent hypoxemia/reoxygenation, the core pathological feature of OSAS [40], can activate the sympathetic nervous system and the adaptive pathway of inflammation, which is the main cause of elevated blood pressure [14, 41, 42]. The AHI as a risk factor for hypertension is consistent with previous findings. Wang et al. [43] found that the AHI was not only positively correlated with the prevalence of hypertension but also significantly correlated with daytime blood pressure. Another cross-sectional study suggested that a one-unit increase in the AHI was associated with a 1% increase in the risk of hypertension [44]. Therefore, it is reasonable to include age, BMI, and the AHI in the prediction model of hypertension.

The presence of nocturnal hypoxemia, inflammation, fragmented sleep, and psychological stress [45] can lead to metabolic and endocrine dysfunction in patients with OSAS, which may result in changes of biochemical parameters. Therefore, the results of routine laboratory tests should also be considered. We found that FBG was a risk factor for hypertension, while HDL-C was a protective factor. Consistent with these findings, the PREVEND prospective cohort study demonstrated that HDL-C was inversely and independently associated with incident hypertension [46]. An increased HDL-C may also indicate the absence of carotid atherosclerosis [47]. A major mechanism underlying hypertension is endothelial dysfunction due to long-term toxic exposure of endothelial cells to proatherogenic lipoprotein components [48]. HDL-C may have a protective effect against hypertension by reducing vascular damage through its antioxidant and anti-inflammatory effects [49]. It also has antithrombotic activity and anti-apoptotic ability and promotes reverse cholesterol transport, which can remove cholesterol in atherosclerosis [50, 51]. Studies of populations in different countries [52–54] have shown that increased FBG is associated with a higher probability of hypertension. High glucose can disrupt endothelial and smooth muscle cell homeostasis by stimulating the formation of advanced glycosylation end products, activating protein kinases, and generating cytotoxic oxygen radicals, thereby increasing vascular stiffness and promoting vascular disease [55]. Insulin resistance, often accompanied by hyperglycemia, can promote proinflammatory cytokine secretion and may accelerate renal tubule reabsorption of sodium and water, resulting in increased systemic sympathetic tone and elevated blood pressure [56].

In addition, our regression analysis showed that TB was one of the more important predictors of hypertension in patients with OSAS. Wang and Bautista [57] analyzed data from the NHANES (*N* = 31,069) and showed that systolic blood pressure was reduced by 1.67 mmHg and that the likelihood of hypertension was reduced by 14% in individuals with TB ≥ 0.7 mg/dl, which was similar to our results. The protective effect could be attributed to antioxidant properties and the ability to maintain the homeostatic vasodilation of bilirubin in the physiological range [58, 59]. However, some research results are not completely consistent with those of our study. For example, a prospective study suggested TB levels above 12.17 μmol/L showed a promoting effect on hypertension [60]. The harmful effects of excessive TB on health may be related to its cytotoxicity [61]. The slight inconsistencies between the results may be due to differences in study subjects and analytical methods. The oxidative stress state in OSAS itself consumes part of TB with antioxidant function, resulting in less individuals with high TB levels in this study.

In summary, this set of valid and concise variables was selected for synthesis in our research model. Then, we visualized the model and represented it as a nomogram. In addition, we developed a scoring system based on the model to assess the risk of hypertension. We compared the prediction model and the scoring system and found that there was no statistical difference in their ability to predict disease risk. They both had moderate discriminative ability and good calibration ability. This study included predictive variables generally consistent with the results of most domestic and foreign studies on hypertension prediction models [62, 63]. For instance, age, sex, systolic blood pressure, BMI, diabetes, cardiovascular disease status, and self-reported total physical activity time were included in the model for predicting hypertension risk in a Canadian population [64]. The Canadian model’s C-statistic (discriminative performance) was 0.77, which is slightly better than our results. However, the Canadian study included a self-reported survey and screened for cardiovascular disease, which was likely to be biased and added to the workload. In contrast, our model consisted of only objective parameters. In terms of clinical feasibility, the few parameters specific to our model were easy to collect during the process of routine clinical care and were inexpensive. In addition, existing hypertension screening tools, most of which have been validated in the general population, have been poorly studied in disease-specific populations, and their sensitivity and specificity are unsatisfactory. For instance, the AUC of a risk prediction model for hypertension in older patients with nonalcoholic fatty liver disease was 0.707 (95% CI: 0.688–0.727) [65], which is similar to that of our model. To the best of our knowledge, this may be the first study to establish a specific model for predicting the risk of hypertension in an older Chinese population with OSAS.

Coupled with the low recognition rate, both hypertension and OSAS can cause a variety of diseases and even fatal events [66, 67], therefore, it is essential to identify hypertension in those patients with OSAS. The intuitive characteristic of the nomogram and the scoring system developed in this study will allow clinicians to make fast and advisable decisions. After screening, for patients who are indicated to have a higher risk of developing hypertension, we recommend interventions that target relevant factors, such as continuous positive airway pressure [68] and hypoglycemic and blood lipid reduction treatment to prevent and cure vascular elastic injury and maintain the function of vital organs. The results of our study will suggest the risk of hypertension in patients with OSAS, help clinicians pay attention to the patients’ systemic conditions for further diagnostic confirmation, and have certain reference value for the selection of treatment methods and the improvement of prognosis of OSAS-related hypertension.

There are a few limitations in this study. First, a model developed based on the Chinese population may not apply to other races. Second, this study only conducted internal validation, and it is hoped that in future studies, the model will be applied to a community population for screening to replenish and improve the external validation and evaluate the performance of the model. Third, considering the deviation of subjective responses, the influences of family history of hypertension and relevant drugs such as hypotensor on OSAS were not analyzed, which could be supplemented by a detailed inquiry plan. Fourth, not all subjects had been objectively tested to rule out the cause of secondary hypertension using only available medical records, so it could not be completely excluded that some individuals were accompanied by other factors causing hypertension. Finally, this study only involved a cross-sectional analysis. To improve the prediction accuracy, the next step is to prospectively observe changes in blood pressure over time in this population, especially in non-hypertensive patients with OSAS.

## Conclusion

For OSAS patients with hypertension risk, we suggest that medical personnel and each individual patient should strengthen the monitoring of six readily available indicators: age, BMI, AHI, FBG, TB, and HDL-C. The nomogram and score established and verified in this study are simple and intuitive to present the risks involved. The results provide a rapid and an accessible reference for reliably identifying patients with OSAS at high risk of hypertension and could be applied to outpatient and community populations for screening and clinical priority treatment. Further large-scale studies are needed to corroborate the effectiveness of the developed methods.

## Supplementary Information

Below is the link to the electronic supplementary material.

Supplementary file1 (DOCX 18 KB)

## Data Availability

The data that support the findings of this study are available on request from the corresponding author or first author. The data are not publicly available due to privacy or ethical restrictions.
