# Supplementary material for: Further insights into influence factors of hypertension in older patients with obstructive sleep apnea syndrome: a model based on multiple centers
Source: Aging Clin Exp Res. 2025 Mar 27;37(1):108. doi: 10.1007/s40520-025-02986-w (PMC11950130; doi:10.1007/s40520-025-02986-w)
Supplement: Supplementary file 1 — Supplementary file1 (DOCX 18 KB) [file 40520_2025_2986_MOESM1_ESM.docx]

Supplementary Table 1 Logistic regression analysis was used to analyze the influencing factors of OSAS related hypertension^&^

|  | *All subjects* | *Male* | *Female* | *60-80 years* | *≥80 years* | *BMI＜28kg/m^2^* | *BMI≥28kg/m^2^* |
| --- | --- | --- | --- | --- | --- | --- | --- |
| *Age* | 1.039^**^  （1.010-1.069） | 1.022^#^  （0.991-1.053） | 1.060^*^  （1.010-1.112） | 1.031^#^  （0.997-1.067） | 1.045^#^  （0.887-1.231） | 1.038^**^  （1.010-1.067） | 1.001^#^  （0.935-1.072） |
| *BMI* | 1.100^**^  （1.042-1.161） | 1.119^**^  （1.047-1.196） | 1.128^**^  （1.049-1.213） | 1.126^△^  （1.070-1.184） | 1.127^#^  （0.944-1.345） | 1.099^*^  （1.021-1.184） | 0.946^#^  （0.793-1.128） |
| *AHI* | 1.012^*^  (1.002-1.022) | 1.003^#^  （0.992-1.015） | 1.015^*^  （1.001-1.030） | 1.007^#^  （0.998-1.017） | 1.010^#^  （0.977-1.045） | 1.012^*^  （1.001-1.022） | 0.997^#^  （0.980-1.015） |
| *FBG* | 1.139^*^  (1.011-1.284) | 1.134^#^  (0.990-1.299) | 1.126^#^  (0.948-1.339) | 1.157^*^  (1.034-1.294) | 0.849^#^  （0.551-1.308） | 1.182^**^  (1.043-1.339) | 1.020^#^  (0.839-1.240) |
| *TB* | 0.952^**^  (0.920-0.985) | 0.954^**^  (0.924-0.986) | 0.958^#^  (0.907-1.011) | 0.957^**^  (0.930-0.984) | 0.944^#^  （0.841-1.060） | 0.962^*^  (0.930-0.994) | 0.935^*^  (0.887-0.985) |
| *HDL-C* | 0.341^△^  (0.195-0.596) | 0.537^*^  (0.293-0.984) | 0.208^△^  (0.086-0.501) | 0.362^△^  (0.216-0.606) | 0.705^#^  （0.100-4.968） | 0.447^**^  (0.250-0.800) | 0.274^**^  (0.108-0.700) |

^&^adjusted for age, gender, BMI, LSpO_2_, total bilirubin, HDL-C, uric acid, creatinine, FBG, homocysteine, hemoglobin, carotid atherosclerosis and CHD. The results were presented as OR (95%CI) in the above table; ^#^*P*>0.05, ^*^*P*<0.05, ^**^*P*<0.01, ^△^*P*<0.001; BMI: body mass index; AHI: the apnea-hypopnea index; FBG: fasting blood glucose; TB: total bilirubin; HDL-C: high density lipoprotein cholesterol.


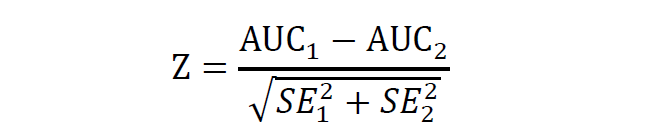


Supplementary Figure 1 The formula for Z-test

(SE_1_ and SE_2_ represented the standard errors of AUC_1_ and AUC_2_, respectively.)

Supplementary Table 1 showed the OR (95%CI) corresponding to each of the contributing factors of hypertension in the different subgroups.

Supplementary Figure 1 showed a universal formula for comparing the discrimination between the model and the score.
